# Identification of key genes and pathways in schizophrenia: a bioinformatics analysis based on GWAS and GEO

**DOI:** 10.3389/fpsyt.2025.1464676

**Published:** 2025-04-07

**Authors:** Alireza Shokrgozar, Maryam Rahimi, Shahrzad Shoraka

**Affiliations:** 1 Department of Psychology, Ka.C., Islamic Azad University, Karaj, Iran; 2 Clinical Cares and Health Promotion Research Center, Ka.C., Islamic Azad University, Karaj, Iran; 3 Department of Microbiology and Microbial Biotechnology, Faculty of Life Sciences and Biotechnology, Shahid Beheshti University, Tehran, Iran

**Keywords:** schizophrenia, bioinformatics analysis, GWAS, GEO, hub gene analysis, key pathway and genes

## Abstract

**Introduction:**

Schizophrenia is a mental illness that is associated with many disorders, such as incoherence of mental activities, and impairment of perception, thinking, emotions, and behavior. Although the exact cause of schizophrenia is unknown, many studies have highlighted the role of genetic background and environmental factors in this disease. Therefore, the identification of key genes involved in schizophrenia provides a promising opportunity to develop novel diagnosis and/or treatment methods. This study aims to investigate schizophrenia-related hub genes by bioinformatics analysis based on genome-wide association (GWAS) and gene expression omnibus (GEO) datasets.

**Material and methods:**

In this study, the GWAS catalog and GEO dataset were used to identify key candidate genes and pathways that are important in the diagnosis and treatment of schizophrenia, and then the results were analyzed using Enrichr and Cytoscape tools.

**Result:**

According to our result NRXN, CACNA1C, and GRIN2A genes had the highest scores in the GWAS analyses and BRCA1, ATM, and STAT1 genes had the highest scores in the GEO dataset. Also, glucuronidation, ascorbate, and aldarate metabolism pathways in the GWAS, PI3K/AKT and Rap1 signaling in the GEO had the highest associations with schizophrenia.

**Conclusion:**

This study highlights the need for further validation of the genes and molecular pathways in schizophrenia. Also, the identified genes could be promising candidates for future diagnostic and/or treatment strategies for schizophrenia.

## 1 Introduction

Schizophrenia is a mental disorder with recurrent psychotic episodes, often characterized by symptoms such as hallucinations, delusions, disordered speech or behavior, and impaired cognitive ability (1). It is estimated that approximately 7 out of every 1,000 people will develop schizophrenia in their lifetime (2). Chronic course and early onset age make schizophrenia a debilitating disease for patients and their relatives (3).

Although schizophrenia is a low-prevalence disorder, it has an important role in the global burden of disease, accounting for about 13 million years of life with disability. Based on clinical and social functioning variables, approximately 1 in 7 cases of schizophrenia recover. However, poor outcomes are common, which include premature mortality, prolonged hospitalization, resistance to treatment, and poor quality of life (4, 5). Although antipsychotics have significantly improved the quality of life in schizophrenic patients, differences in treatment response and tolerance to drugs have been observed among individuals. It is possible that an antipsychotic significantly improves some patients, while others show no response and/or experience adverse side effects to that medication. It is estimated that about 30% of schizophrenic patients experience treatment resistance. On the other hand, the poorly understood pathophysiology of schizophrenia leads to therapeutic stagnation. Selection of an effective and appropriate antipsychotic through the traditional “trial and error” approach poses significant challenges for both the patient and the health care system. Therefore the possible role of genetic factors in the effectiveness and side effects of antipsychotic treatment has drawn the attention of many researchers to pharmacogenetics and personalized medicine in schizophrenia (6, 7).

The interaction between a genetic background and the environment in the development of schizophrenia is well established. Schizophrenia is highly heritable, with an estimated heritability of around 80%. In addition to the genetic background, exposure to various environmental factors during different stages of development (prenatal and perinatal life, adolescence and adulthood) also plays a role in the risk of developing schizophrenia. Some of these environmental factors have cumulative effects and may be interrelated and possibly share common causal pathways. It is therefore likely that the development of schizophrenia is the result of an interaction between genetic and environmental factors, rather than the result of their independent effects. This possibility has led to a significant number of studies to identify environmental and genetic risk factors involved in schizophrenia (8, 9). In addition, recent studies have focused on the role of epigenetics in filling the gap between genetics and the environment (6).

Understanding the interplay between genetic and environmental risk factors in schizophrenia is of great importance, as it allows the identification of disease-related pathways that can be used to identify individuals at higher risk, early diagnosis, prevention programs, clinical management, effective treatment, and also drug development (9, 10).

Although no single gene or variant has been identified as contributing to schizophrenia, some single nucleotide polymorphisms (SNPs) have been implicated in the pathogenesis of the disease with high effect sizes. In recent years, by using large exome and genome sequencing data, it has become possible to identify rare coding variants that are associated with the risk of schizophrenia (9). Since it is estimated that about half to one-third of the genetic risk is indexed by variants identified by genome-wide association (GWAS (arrays, GWAS is a critical method for finding the biological constructs of schizophrenia. In addition, the online GEO (Gene Expression Omnibus) database contains numerous datasets of schizophrenia, including common species such as humans and mice, and bioinformatics analysis of schizophrenia is often based on this database (1, 11). The aim of this study is to investigate schizophrenia-related hub genes and pathways extracted from the GWAS catalog and GEO dataset.

## 2 Material and methods

### 2.1 GWAS catalog study

A list of 3344 SNPs was extracted using the GWAS catalog dataset and results from 80 published studies. Then, the SNPs with *p value* ≤ 5×10 ^-8^ were separated and then the Biomart of the ensemble.org website was used (12). After that, the Biomart results were sorted and collected based on the obtained genes and omitting duplicates, and 554 SNPs were found. For this process; this inclusion and exclusion criteria as applied:

1. Study Design: Only genome-wide association studies (GWAS) focusing on schizophrenia were considered.
2. Significance Threshold: Studies reporting SNPs with a genome-wide significance threshold (p ≤ 5 × 10^-8^) were included.
3. Population Diversity: Studies including diverse ethnic populations were considered to enhance generalizability.
4. Sample Size: Studies with a sufficiently large sample size (n ≥ 500 cases) to ensure statistical power were included.
5. Replication Studies: Studies that validated their findings through independent replication cohorts were preferred.

**Exclusion Criteria:**

1. Non-GWAS Studies: Studies that did not conduct a genome-wide association analysis were excluded.
2. Lack of Statistical Significance: Studies that did not provide genome-wide significant SNPs (p > 5 × 10^-8^) were not included.
3. Redundant or Overlapping Data: Studies with overlapping cohorts without independent validation were removed to avoid redundancy.

The list of 339 gene names obtained from Biomart tool was entered into the Enrichr database for enrichment analysis.

Also, the list of genes was entered into Cytoscape software (13, 14) and checked with CluePedia (15) and Clue GO tools (Pathways = GO Biological process, GO Cellular component, GO Molecular function) (16).

Genes with a *p value* ≤ 0.05 were considered significant and were analyzed. CluePedia analyzed 250 of the lists of all genes and showed 2357 results. CluePedia results were separated, sorted and checked based on GO term, Average Short Path Length, Betweenness Centrality, Closeness Centrality, Clustering Coefficient, Degree, Eccentricity, and Neighborhood Connectivity columns (Results were rechecked with Enrichr).

The protein-protein interactions (PPIs) network was investigated and visualized using Cytoscape software. The network was then evaluated using the NetworkAnalyzer plugin, with metrics like Degree, and Neighborhood Connectivity to identify the hub genes/proteins. In our study, hub genes were identified using the Degree Centrality method within the NetworkAnalyzer plugin in Cytoscape. Degree Centrality measures the number of direct connections (edges) a node (gene/protein) has within the network, reflecting its influence and importance (17–19).

### 2.2 GEO study

A microarray dataset (GSE92538) was selected from the GEO database (20). GSE92538 dataset includes human clinical trials that predicted the relative cell type composition for 157 human dorsolateral prefrontal cortex samples based on Affymetrix microarray data collected by the Pritzker Neuropsychiatric Consortium, as well as for 841 samples spanning 160 brain regions using an Agilent microarray platform collected by the Allen Brain Atlas. 119 healthy controls and 27 samples of schizophrenic patients were analyzed with the GEO2R tool (21), which resulted in 1763 cases remaining for analysis.

Also, the protein-protein interactions (PPIs) network was created utilizing Cytoscape software. The network was assessed using the NetworkAnalyzer plugin, and hub genes were identified using the Degree Centrality method within the network.

## 3 Results

### 3.1 Pathway, GO, and disease enrichment analysis based on GWAS study

339 genes were entered and analyzed in the Enrichr tool. All enrichment with an adjusted *p value* less than 0.05 was considered statistically significant.

As shown in **Tables 1** and **2**, the key metabolic pathways are glucuronidation, ascorbate and aldarate.

**Table 1:** Pathways of GWAS catalog genes (KEGG).

Index	Name	P-value	Gwasted p-value	Odds Ratio	Combined score
1	Ascorbate and aldarate metabolism	5.500e-7	0.0001183	18.00	259.47
2	Pentose and glucuronate interconversions	0.000001371	0.0001474	15.33	206.98
3	Porphyrin and chlorophyll metabolism	0.000007206	0.0005164	11.49	136.10
4	Retinol metabolism	0.00001914	0.001029	7.90	85.78
5	Steroid hormone biosynthesis	0.00007514	0.003231	7.66	72.70

**Table 2:** Pathways of GWAS catalog genes (WIKI pathways).

Index	Name	P-value	Adjusted p-value	Odds Ratio	Combined score
1	Glucuronidation WP698	1.884e-7	0.00006330	21.80	337.52
2	Codeine And Morphine Metabolism WP1604	0.0001256	0.01467	19.55	175.62
3	NRXN1 Deletion Syndrome WP5398	0.0001621	0.01467	18.05	157.50
4	Prion Disease Pathway WP3995	0.0001878	0.01467	10.89	93.41
5	Disruption Of Postsynaptic Signaling By CNV WP4875	0.0002182	0.01467	10.50	88.49

Gene Ontology analysis showed that the genes associated with the biological pathways of retinoic acid binding and monocarboxylic acid binding are the significant terms based on the adjusted p-value, respectively (**Table 3**).

**Table 3:** Gene Ontology of GWAS catalog genes (Molecular function).

Index	Name	P-value	Adjusted p-value	Odds Ratio	Combined score
1	Retinoic Acid Binding (GO:0001972)	1.598e-8	0.000005945	34.52	619.77
2	Monocarboxylic Acid Binding (GO:0033293)	3.293e-7	0.00006126	19.72	294.33
3	Retinoid Binding (GO:0005501)	5.500e-7	0.00006820	18.00	259.47
4	Glucuronosyltransferase Activity (GO:0015020)	0.000009554	0.0008886	14.74	170.40
5	Hexosyltransferase Activity (GO:0016758)	0.0002278	0.01695	5.38	45.09

Gene-disease association analysis was also performed with Enrichr, shown in **Tables 4**–**6**. The results showed that genes are associated with schizophrenia and related diseases such as bipolar and schizoaffective disorders and especially treatment with antipsychotic drugs in schizophrenia patients.

**Table 4:** Disease of GWAS catalog genes (dbGaP).

Index	Name	P-value	Adjusted p-value	Odds Ratio	Combined score
1	Schizophrenia	1.125e-20	2.341e-18	22.67	1041.41
2	Blood Pressure	1.478e-14	1.537e-12	5.47	174.18
3	DNA	1.321e-11	9.162e-10	207.25	5191.53
4	Anemia, Sickle Cell	2.782e-10	1.447e-8	82.89	1823.72
5	Echocardiography	1.743e-9	6.706e-8	5.37	108.24

**Table 5:** Disease of GWAS catalog genes (GWAS catalog).

Index	Name	P-value	Adjusted p-value	Odds Ratio	Combined score
1	Schizophrenia	1.929e-262	4.945e-259	73.65	44381.73
2	Cognitive Ability, Years of Educational Attainment or Schizophrenia (Pleiotropy)	2.004e-186	2.568e-183	275.69	117879.66
3	Schizophrenia (MTAG)	3.542e-112	3.026e-109	164.80	42291.40
4	Educational Attainment (MTAG)	2.208e-77	1.415e-74	15.08	2662.19
5	Educational Attainment (Years of Education)	1.261e-76	6.466e-74	14.41	2518.37

**Table 6:** Disease of GWAS catalog genes (DisGeNET).

Index	Name	P-value	Adjusted p-value	Odds Ratio	Combined score
1	Schizophrenia	1.846e-97	7.033e-94	12.23	2725.00
2	Autism Spectrum Disorders	6.244e-43	1.190e-39	10.54	1024.13
3	Intelligence	2.837e-41	3.602e-38	19.96	1863.96
4	Cognition	4.164e-22	3.966e-19	36.19	1781.75
5	Systolic Pressure	9.926e-15	7.564e-12	12.52	403.55

### 3.2 Protein–protein interaction network and hub genes analysis

**Figure 1:**
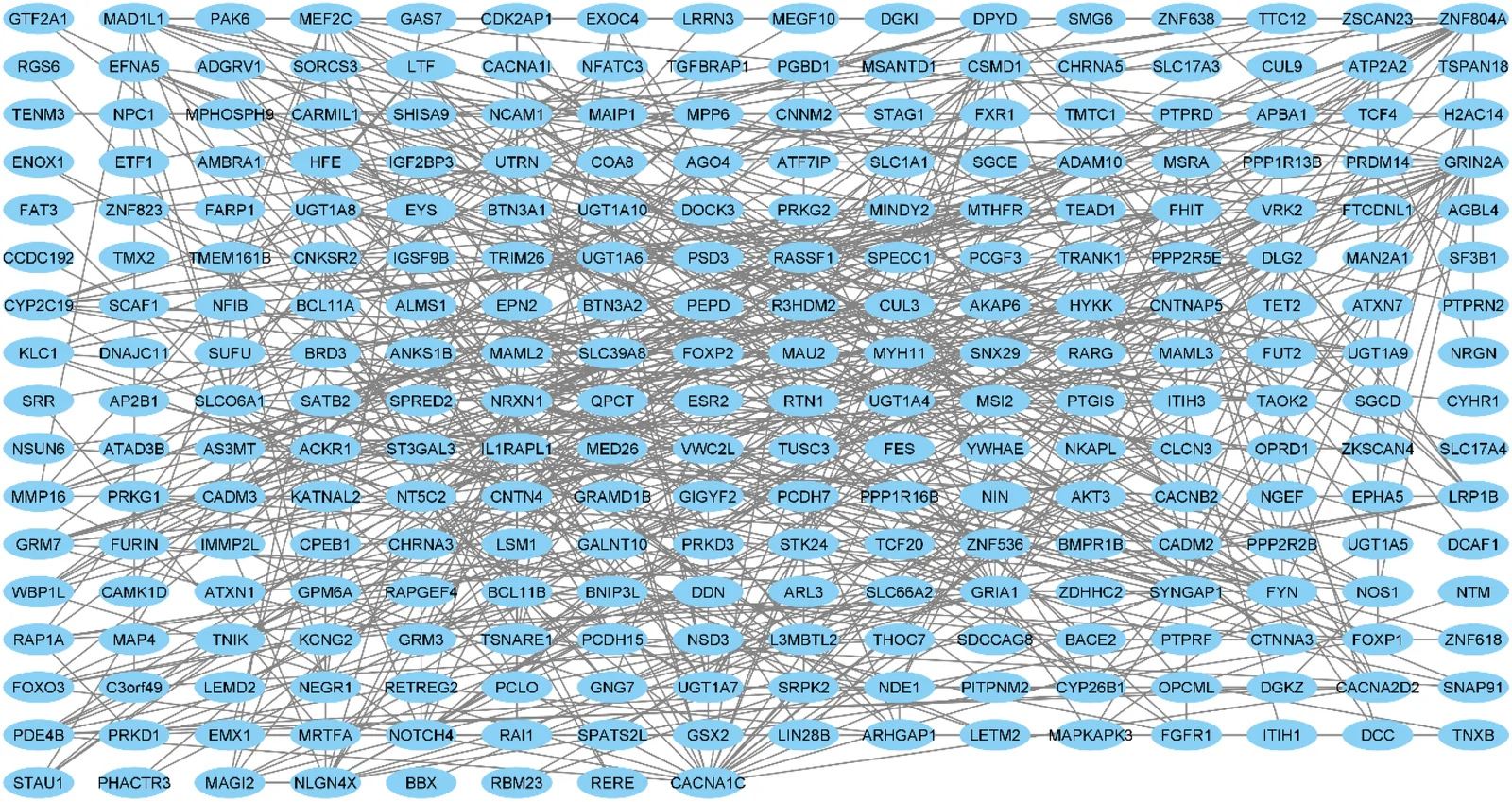
Interactions between GWAS catalog genes.

The core genes were identified in Cytoscape software and the results showed that NRXN, CACNA1C and GRIN2A are hub genes in schizophrenia. The results are shown in **Figure 1** and **Table 7**.

**Table 7:** Top 10 hub genes ranked by the degree centrality method (GWAS catalog database).

Rank	Name	Score
1	NRXN1	27
2	CACNA1C	26
3	GRIN2A	22
4	ZNF804A	19
5	GRIA1	18
6	FYN	16
6	CACNB2	16
8	DPYD	14
8	CSMD1	14
8	DLG2	14

### 3.3 Pathway, GO, and disease enrichment analysis based on GEO Study

95 genes were entered and analyzed in the Enrichr database. All enrichment with an adjusted *p value* below 0.05 was considered statistically significant.

As shown in **Table 8**, PI3K/AKT and Rap1 signaling pathways can be considered as the main pathways in schizophrenia.

**Table 8:** Pathways of GEO gene.

Index	Name	P-value	Adjusted p-value	Odds Ratio	Combined score
1	PI3K-Akt signaling pathway	1.269e-8	0.000002386	9.10	165.38
2	Pathways in cancer	0.000001345	0.0001264	5.93	80.22
3	Rap1 signaling pathway	0.000007185	0.0004503	8.97	106.23
4	Ras signaling pathway	0.00001483	0.0006972	8.08	89.83
5	Measles	0.00005291	0.001990	10.02	98.69

GO analysis showed that protein serine/threonine kinase activity has the highest OR, shown in **Table 9**.

**Table 9:** Gene Ontology of GEO genes (Molecular function).

Index	Name	P-value	Adjusted p-value	Odds Ratio	Combined score
1	Protein Serine/Threonine Kinase Activity (GO:0004674)	0.00003634	0.004080	6.15	62.88
2	DNA Binding (GO:0003677)	0.00004459	0.004080	3.96	39.69
3	Cytokine Activity (GO:0005125)	0.0002061	0.01257	7.73	65.64
4	Receptor Ligand Activity (GO:0048018)	0.0008191	0.03554	5.00	35.50
5	Translation Initiation Factor Activity (GO:0003743)	0.0009710	0.03554	17.05	118.27

### 3.4 Protein–protein interaction network and hub genes analysis

**Figure 2:**
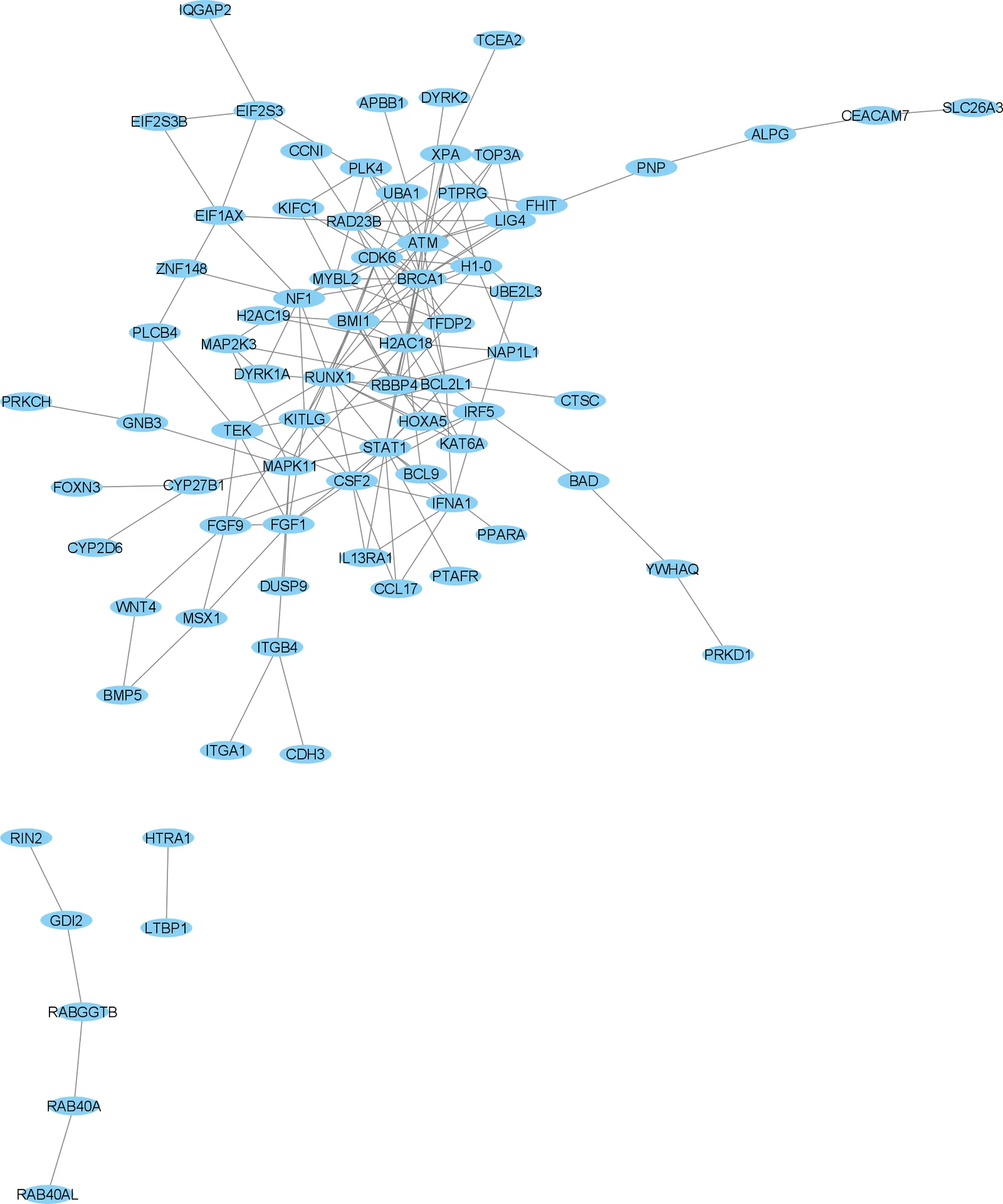
Interactions between GEO genes.

As shown in **Figure 2** and **Table 10**, BRCA1, ATM and STAT1 genes are the hub genes with the highest score in Cytoscape software.

**Table 10:** Top 10 hub genes ranked by the degree centrality method (GEO database).

Rank	Name	Score
1	BRCA1	21
2	ATM	17
3	STAT1	15
4	RUNX1	13
4	BMI1	13
6	H2AC18	12
7	CSF2	11
7	CDK6	11
9	BCL2L1	10
10	NF1	9

## 4 Discussion

The mechanism of schizophrenia is still not fully understood. Therefore, understanding the key genes and pathways in schizophrenia can help in early diagnosis, prevention and control programs, patient management, and treatment selection as well as the development of new drugs (9).

In the present study, the data obtained from the GEO and GWAS databases were analyzed to discover the key genes and pathways involved in schizophrenia.

### 4.1 GWAS study

In recent years, GWAS has been developed and has led to many findings, which are presented as GWAS catalogs (22). According to our findings from GWAS, the key pathways related to schizophrenia were glucuronidation, ascorbate and aldarate metabolism. Glucuronidation is a detoxification process or a defense mechanism involved in removing unwanted substances such as endogenous compounds, drugs, and other xenobiotics from the body (23). Previous studies have also shown that drug resistance in schizophrenia is associated with increased expression levels and polymorphism of proteins responsible for drug glucuronidation (24). Clozapine (CLZ) is the only FDA-approved antipsychotic for the treatment of drug-resistant schizophrenia, and glucuronidation plays a key role in its metabolism. Various studies have shown the relation between the glucuronidation pathway and CLZ. Also, several studies have shown the relationship between polymorphisms in active glucuronidation enzymes and changes in CLZ metabolism (25, 26). A GWAS study in 2019 reported the association between UDP-glucuronosyltransferase polymorphisms and CLZ plasma concentration in schizophrenic patients (27).

The next key schizophrenia-related pathway found in this study was ascorbate and aldarate metabolism, which in previous studies have been reported to be biologically or molecularly associated with psychiatric disorders, including bipolar disorder and dementia (28). Similar to our results, recent studies reported that ascorbate and aldarate metabolism pathways are enriched in schizophrenic patients (29). Also, a 2023 shotgun metagenomic study of gut microbiota that included patients with schizophrenia and healthy controls showed that ascorbate and aldarate metabolism are enriched in patients (30). Similarly, a systematic review of gut microbiota composition reported enriched ascorbate and aldarate pathway in schizophrenic patients (50%; N = 2/4 reported studies) (31). Ascorbate and aldarate metabolism is an important carbohydrate metabolic pathway that plays a role in protecting cells from oxidative damage (32). Considering the possible role of oxidative stress in the pathophysiology of schizophrenia, many studies have reported the administration of antioxidants including vitamin C (Ascorbate) to be effective in improving the negative symptoms in schizophrenic patients (33, 34).

The results of GO analysis demonstrated that retinoid acid binding and monocarboxylic acid binding have the highest OR respectively. Considering that retinoid signaling has an important role in the function of immune cells, therefore factors affecting it can have significant consequences for the inflammatory stress associated with schizophrenia. Biochemical and clinical evidence of schizophrenia supports retinoid dysregulation. It has been suggested that clozapine modulates retinoid homeostasis in the brain and also normalizes serum retinoic acid deficiency in schizophrenic patients. In addition, several SNPs related to retinoids have been reported to be associated with schizophrenia (35, 36). Therefore, the use of a retinoid agonist (bexarotene) has been suggested as an additional therapeutic agent for the treatment of symptoms in schizophrenic patients (37).

As mentioned earlier, our GO analysis showed that monocarboxylic acid binding was also enriched. Carboxylic acids comprise a large and heterogeneous group of endogenous and exogenous compounds that are involved in several drugs, including antipsychotics such as gabapentin and clozapine, which are used to treat schizophrenia (38).

Based on disease enrichment analysis, we found that there is an association between genes with schizophrenia and other diseases such as bipolar and schizoaffective disorders, and especially antipsychotic drug treatment in schizophrenic patients. This is very important because choosing an effective and appropriate drug for each patient with schizophrenia is vital to controlling symptoms (39). Pharmacogenetic research has focused on identifying genetic variants that contribute to individual variation in several antipsychotic-related phenotypes. Recent studies have been successful in identifying genetic variants associated with antipsychotic treatment (40, 41). In this regard, several genes have been found related to the response to antipsychotic drugs, in which genetic variation can lead to differences in effectiveness and side effects. Most research focuses on SNPs obtained throughout GWAS or candidate gene studies. It has been suggested that such an approach could help predict the success of treatment with antipsychotic drugs (42).

Also, our PPI network analysis identified NRXN, CACNA1C, and GRIN2A as hub genes in schizophrenia. Norexins are cell surface receptors that bind neuroligins to form Ca(2+)-dependent neuroxin/neuroligin complexes at central nervous system (CNS) synapses. Among NRXN genes, NRXN1 is involved in neurodevelopmental and psychiatric disorders. Another GWAS studies have consistently found associations between copy number variation (mainly deletions) in NRXN1 and schizophrenia (43, 44).

The protein encoded by the CACNA1C gene is the component of the calcium channel. Consistent with our study, the CACNA1C gene has been suggested as a risk factor for schizophrenia in a GWAS study (45). In addition, a recent meta-analysis study reported rs1006737, rs2007044 and rs4765905 of the CACNA1C gene to be associated with susceptibility to schizophrenia (46).

The protein encoded by the GRIN2A gene is a member of the glutamate-gated ion channel family. Previous studies have shown that dysfunction of GRIN2A is probably important in the development of schizophrenia (47). A study conducted in 2023 showed that there is an association between rs11644461 GRIN2A and the clinical phenotype of schizophrenia. They reported that TC rs11644461 GRIN2A genotype carriers have higher severity of schizophrenia symptoms than CC genotype carriers (48).

### 4.2 GEO study

In this study, we also analyzed the differentially expressed genes from the GEO dataset. The pathway analysis showed that the main pathways in schizophrenia were PI3K/AKT signaling and Rap1 signaling. The PI3K/AKT pathway can serve as a central intracellular network for the action of synaptic neuroplasticity. In neural tissue, mutations on this pathway lead to a different phenotype that affects neuronal morphology and synaptic transmission, and in some cases, severe learning and behavioral imbalances. A link between altered PI3K/AKT signaling and schizophrenia pathogenesis has been suggested (49). Recently, pharmacological targeting of PI3K or related factors has been considered a novel promising tool for the treatment of schizophrenia (50). Since some diets may contribute to neuro-protection in psychiatric diseases through modulation of PI3K/AKT/GSK3 signaling, developing treatments for mental disorders with a nutritional approach is challenging (51).

The next schizophrenia-related pathway was the Rap1 signaling pathway, which plays a role in synaptic plasticity, excitability, learning and memory by inhibiting the release of L-type calcium channel-dependent neurotransmitters (52). Previous studies have reported that cAMP-dependent protein kinase and one of its substrates, Rap1, are altered in patients with affective disorders. Furthermore, abnormalities in cAMP-dependent protein kinase have been reported in platelets from patients with obsessive-compulsive disorder (OCD) and schizophrenia (53). A recent study showed that there is no difference in Rap1 levels between patients with OCD and schizophrenia patients, while the phosphorylation status of Rap1 in OCD groups is significantly lower than that of schizophrenic patients and the control group (53). Similar to our results, analysis of schizophrenia-related genes through a pathway and network-based approach reported the Rap1 signaling pathway significantly enriched in schizophrenia (52).

The results of GO analysis also revealed that protein serine/threonine kinase activity was one of the highest OR. Serine/threonine kinases play a key role in regulating cell proliferation, cell differentiation, apoptosis, and embryonic development. Several studies have shown a link between serine/threonine kinase and schizophrenia. A 2014 study showed that substrate peptides are differentially phosphorylated in schizophrenia and comparison groups. They reported that 14 peptides showed a 1.15-fold or greater increase in kinase activity, while 5 peptides had a 1.15-fold or greater decrease. Investigating kinase activity in signaling pathways can be effective in identifying new platforms for discovering safe and effective drugs for schizophrenia (54).

Our disease enrichment analysis demonstrated that deletion of activity-dependent synaptic AMPA receptors (AMPARs) had the highest OR. AMPARs mediate almost all fast excitatory neurotransmission in the mammalian CNS. Also, the prominent role of AMPARs in mediating glutamatergic synaptic transmission and plasticity has been identified (55). AMPA receptor-positive modulators can improve cognitive performance in schizophrenia, and their enhancement of AMPA receptor-mediated currents enhances the activity of antipsychotic drugs (56).

In the PPI network, we found three hub genes including BRCA1, ATM, and STAT1. The BRCA1 is a known tumor suppressor gene that plays an important role in protecting genomic stability. A study conducted in 2016 to identify gene expression abnormalities in schizophrenia examined whole-genome gene expression profiles using microarrays of peripheral blood mononuclear cells (PBMCs). Consistent with our study, they introduced BRCA1 as a hub gene. They also reported that BRCA1 is upregulated in schizophrenia patients compared to the healthy group. Their findings suggested that mRNA co-expression abnormalities may act as a promising mechanism in the development of schizophrenia (57).

The ATM gene is one of the most important genes in the DNA repair pathway, and by identifying and responding to DNA damage, it leads to the stability of the genome. A recent study suggested the ATM gene as a possible candidate for schizophrenia susceptibility. The variant G allele of the rs609429 polymorphism may provide further insight into the association of schizophrenia with genetic background. It has been suggested that individuals carrying the G allele have lower expression levels and, consequently, lower DNA repair capacity compared to individuals carrying the C allele, and are more susceptible to schizophrenia (58).

Activation of STAT1 is downstream of cytokine receptors that signal from specific pro-inflammatory cytokines known to be dysregulated in schizophrenia, such as IFNγ, IL-6, IL-2, and IL-10. A previous study suggested that activated phosphorylated STAT1 levels may provide a measure of the biological relevance of increased cytokine levels in schizophrenia (59).

## 5 Limitations

Our study sheds light on important genes and pathways involved in schizophrenia, but it has some limitations that should be considered.

### 5.1 Dataset Selection and Potential Bias

Since we relied on publicly available GWAS and GEO datasets, our findings may be influenced by differences in sample selection, diagnostic criteria, and population diversity. These factors could introduce bias, making it important to validate results in larger and more diverse cohorts.

### 5.2 Choice of Tools and Alternative Approaches

The tools we used—Cytoscape’s NetworkAnalyzer, Enrichr, and Biomart—are widely accepted in bioinformatics, but different tools or methods (e.g., machine learning or alternative network analysis techniques) could yield varying results.

### 5.3 Connection to Schizophrenia Models

Our findings highlight genes involved in dopaminergic, glutamatergic, and calcium signaling pathways, which align with existing schizophrenia hypotheses. More functional studies are needed to clarify these relationships.

## 6 Conclusion

In conclusion, our study demonstrated that genetic variations, including single nucleotide polymorphisms, play an important role in the development of schizophrenia. These research findings can be potential candidates to better understand the pathophysiology of schizophrenia and identify new diagnostic and/or therapeutic targets.

The genes and pathways identified in this study could be important and deserve further investigation. It is suggested to evaluate the genes from this study in clinical samples of schizophrenic patients to identify possible biomarkers and also to determine the most important genes in *in vivo* studies for use in treatment as well as compare these results with other mental diseases.

## Data Availability

The original contributions presented in the study are included in the article/supplementary material. Further inquiries can be directed to the corresponding author.
