# Correction: Identification of key genes and pathways in schizophrenia: a bioinformatics analysis based on GWAS and GEO

**DOI:** 10.3389/fpsyt.2026.2003762

**Published:** 2026-10-02

**Authors:** Alireza Shokrgozar, Maryam Rahimi, Shahrzad Shoraka

**Affiliations:** 1 Department of Psychology, Ka.C., Islamic Azad University, Karaj, Iran; 2 Clinical Cares and Health Promotion Research Center, Ka.C., Islamic Azad University, Karaj, Iran; 3 Department of Microbiology and Microbial Biotechnology, Faculty of Life Sciences and Biotechnology, Shahid Beheshti University, Tehran, Iran

**Keywords:** schizophrenia, bioinformatics analysis, GWAS, GEO, hub gene analysis, key pathway and genes

The affiliations were incorrectly published and have been corrected.

The affiliations incorrectly stated:

“1 Department of Psychology, Islamic Azad University, Karaj, Iran, 2Clinical Care and Health Promotion Research Center, Islamic Azad University, Karaj, Iran, 3Department of Microbiology and Microbial Biotechnology, Faculty of Life Sciences and Biotechnology, Shahid Beheshti University, Tehran, Iran”

The affiliations have been corrected to:

“1 Department of Psychology, Ka.C., Islamic Azad University, Karaj, Iran.

2 Clinical Cares and Health Promotion Research Center, Ka.C., Islamic Azad University, Karaj, Iran.

3 Department of Microbiology and Microbial Biotechnology, Faculty of Life Sciences and Biotechnology, Shahid Beheshti University, Tehran, Iran”

The original article has been updated.

